# Quality Properties of Sesame and Olive Oils Incorporated with Flaxseed Oil

**DOI:** 10.15171/apb.2017.012

**Published:** 2017-04-13

**Authors:** Fataneh Hashempour-Baltork, Mohammadali Torbati, Sodeif Azadmard-Damirchi, Geoffrey Peter Savage

**Affiliations:** ^1^Department of Food Science and Technology, Faculty of Nutrition, Tabriz University of Medical Sciences, Tabriz, Iran.; ^2^Department of Food Science and Technology, Faculty of Agriculture, University of Tabriz, Tabriz, Iran.; ^3^Food Group, Department of Wine, Food and Molecular Biosciences, Lincoln University, Canterbury, New Zealand.

**Keywords:** Blending oil, Essential fatty acid, Flaxseed oil, Nutrition, Oxidation stability

## Abstract

***Purpose:*** Suitable ratio of essential fatty acids has an important role in maintaining good health. There is no pure oil with an ideal fatty acid composition and oxidative stability. The main goal of the present study was to evaluate the physical, chemical and nutritional properties of oil obtained by blending flaxseed oil as a rich source of ω_3_ fatty acids with sesame and olive oils.

***Methods:*** Three different ratios (65:30:5, 60:30:10 and 55:30:15) were prepared using olive, sesame and flaxseed oils. These mixtures were stored at 4°C and 24°C and their quality and physicochemical properties were determined by measuring the fatty acid composition, phenolic compound, peroxide, anisidine values and schaal tests.

***Results:*** Fatty acid composition indicated that adding 10% and 15% flaxseed oil into blends have suitable ratio of essential fatty acids. The sample which contained 5% flaxseed oil had the highest phenolic content among treatments and these compounds showed a significant decrease during storage. A significant increase (p<0.05) was observed in peroxide values of all samples during storage. Increasing the flaxseed oil content in the blends, lead to an increase of the anisidine value.

***Conclusion:*** Blending sesame and olive oils with flaxseed oil produced oil blends with a good balance of essential fatty acids. Although peroxide and anisidine values increased during storage of the oil blends; the blends were of a good quality for home and industrial use.

## Introduction

Oils and fats have important roles in cooking, frying and salad dressings or in food formulations; they make an important contribution to our diet and our health. Omega-3 (ω_3_) fatty acids such as linolenic acid, and omega-6 (ω_6_) fatty acids such as linoleic acid, are essential fatty acids; thus, they must be supplied through foods. Today, most of the oils consumed in our daily diet such as corn, sunflower, rice bran and grape seed oils have a high amount of ω_6_fatty acids and therefore, lead to ω_6_:ω_3_ ratio increases ranging from 8-12:1; however, this ratio should be 4:1.^1^

ω_3_ and ω_6_ fatty acids are essential for normal growth and have an important role in the prevention of cancer and cardiovascular diseases as well as the improvement of immune function. The ω_6_ eicosanoids without ω_3_ fatty acids are pro-inflammatory and may lead to high blood pressure, cardiovascular diseases and arthritis.^1^ Therefore, their balance is very important in our daily intake.

Different fats/oils have various chemical and physical properties. Pure vegetable oils can have low functional characterization or nutritional properties. For example, using olive oil or sesame oil alone has some drawbacks such as their low amount of ω_3_ essential fatty acids. Instead, flaxseed oil is a rich source of ω_3_, tocopherols and other bioactive compounds,^2^ but in pure form it is very unstable and oxidizes quickly.

Sesame oil is a good source of ω_6_ fatty acids and has a considerable level of sesamin and sesamolin lignans, which have different bioactive and health promoting effects.^3^ Furthermore, sesame oil has anti-inflammatory activity and antiproliferative effects on cancer cells caused by tocopherol homologues.^4,5^ Sesame oil in spite of containing 85% unsaturated fatty acids, is one of the most stable vegetable oils to oxidation.^6^ However, sesame oil, which has positive nutritious and healthy effects, is low in ω_3_ fatty acids and because of its high price has limited application in the food industry.

Olive oil is a good source of ω_9_ and bioactive compounds such as phenolic compounds and phytosterols^7,8^ and because of its fatty acid composition and natural antioxidants such as tocopherols and polyphenols, it is more stable to heat treatments.^9^

Consuming edible oils with a suitable ratio of essential fatty acids, appropriate stability to heat treatment and storage is a very important issue in the food industry. Unfortunately, no pure oil has both an ideal fatty acid composition and good oxidative stability.

Blending is the simplest physical and economical procedure to change fatty acid composition, increase the bioactive components and natural antioxidants and make a new at an affordable price.^10,11^

Flaxseed and products fortified with flaxseed powder and oil is gaining more attention because of its valuable nutritional properties.^12^ In the present study, flaxseed oil was blended with sesame and olive oils in different ratios to provide optimal essential fatty acids with a suitable stability and high bioactive content. These mixtures were stored at different temperatures to study the feasibility of introducing these functional vegetable oils to the market.

## Materials and Methods

### Materials

The flaxseed and sesame oils were obtained from seeds using a cold press (Screw Press Model 85 mm) and olive oil was purchased from local market (Tabriz, Iran). All chemicals used in this study were of analytical grade and purchased from Sigma Chemical Co (St. Louis, Mo, USA).

### Methods

#### Blending Process

Three oils were prepared using olive: sesame: flaxseed in ratios of 65:30:5, 60:30:10 and 55:30:15 in triplicate. The oil mixtures were stored at 4 and 24°C for 90 days. Experiments were carried out on the production day and every 30 days. The fatty acid profile was determined on the first and 90^th^ days.

#### Fatty Acid Composition

Fatty acid compositions were measured as fatty acid methyl esters by gas chromatograph (GC-1000, DANI, Italy) according to the method described by Savage *et al.*^12^

#### Total Phenolic Compound

Total phenolic content (TPC) was determined using the Folin–Ciocalteau reagent and caffeic acid for calibration curve by spectrophotometer (CECIL, Aquaris 1100, England) at 725 nm according to Capannesi *et al.*^13^ TPC was determined by calibration curve which was achieved from defined concentration of caffeic acid absorbance, and the final results were reported as mg caffeic acid/kg of oil.

#### Stability Tests

Peroxide value (PV) was determined using the AOAC methods.^14^ P-anisidine value (AnV) of the oils was determined by dissolving the oil samples in iso-octane, and the absorbance was measured at 350 nm after 10 minutes by spectrophotometer according to ISO 6885.^15^

#### Schaal test 

The oil samples were placed in a series of 20 ml clear glass bottles incubated in a forced-draft air oven set at 65℃ for 10 days. The oxidation reaction was accelerated in the oil samples, and peroxide value was determined in triplicate after each 24 hours for 10 days.

#### Statistical Analysis

All parameters were measured in triplicate. Data obtained were analyzed by ANOVA using the SPSS statistical software (Chicago, IL, USA) in factorial experiments in completely randomized design, and the results were reported as mean ± standard deviation (SD) of three amounts. Duncan’s multiple range post hoc test was used to analyze significant differences at the 0.05 level.

## Results and Discussion

### Fatty acid composition

Fatty acid composition of edible oils and fats is important from the technological and nutritional points of view. Therefore, one of the main analyses in oils and fats field is fatty acid composition. Results show that fatty acid compositions of flaxseed, sesame and olive oils used in this study are in agreement with previously published data.^16^ Sesame and olive oils had very low amounts of linolenic acid, but flaxseed was a rich source of linolenic acid (Table 1).

**Table 1:** Fatty acid composition (%) of pure olive, sesame and flaxseed oils

**Fatty acid**	**Flaxseed oil**	**Olive oil**	**Sesame oil**
C16:0	6.7	13.3	10.7
C18:0	2.5	4	6.5
C18:1	20.3	69.1	41.8
C18:2	12.9	11.4	40.1
C18:3	57.1	1.2	0.8
ω6:ω3	0.22:1	9.5:1	50.1:1

Blending of sesame and olive oils with flaxseed oil leads to significant changes (p<0.05) in the fatty acid composition (Table 2). Linolenic acid was increased by increasing flaxseed oil in the blend.

A suitable intake of the essential fatty acids is very important in daily diet. According to the literature, the optimal ratio of ω_6_:ω_3_ is reported to range from 1:1 to 4:1.^1^ The ratio of ω_6_:ω_3_ in olive, sesame and flaxseed oils were 9.5:1, 50.1:1 and 0.22:1, respectively (Table 1). This result shows that olive and sesame oils do not have optimal ω_6_:ω_3_ ratios. Therefore, consumers should have other ω_3_ rich oils in their diet for long term usage.

Based on the high content of linolenic acid in flaxseed oil, which is about 50 times higher than olive and sesame oils (Table 1), addition of flaxseed oil to sesame and olive oil mixtures caused an effective decrease in ω_6_:ω_3_ ratio (Table 2), which is very important from nutritional prospective. Therefore, blending flaxseed oil with other vegetable oils which are low in ω_3_ fatty acid can change their fatty acid composition and increase ω_3_ fatty acid content. Samples containing 10% and 15% flaxseed have optimal levels of ω_6_:ω_3_ ratio as stated in the literature for better health achievement (Table 2).^1^

**Table 2:** Fatty acid composition (%) of the three oil blends on the first and 90^th^ day storage at different temperatures.

**Fatty acid**	65:30:5*			**60:30:10**			**55:30:15**		
	**Day1**	**Day 90**		**Day1**	**Day 90**		**Day1**	**Day 90**	
	**25°C**	**4°C**	**25°C**	**25°C**	**4°C**	**25°C**	**25°C**	**4°C**	**25°C**
C16:0	12.1 ± 0.2f	12.2 ± 0.2f	12.5 ± 0.3e	11.60 ± 0.1g	13.9 ± 0.1d	14.0 ± 0.1c	10.6 ± 0.1 h	14.2 ± 0.2 b	14.4 ± 0.4a
C18:0	4.0 ± 0.3h	6.2 ± 0.1f	6.50 ± 0.1e	14.1 ± 0.2g	8.2 ± 0.2d	8.4 ± 0.4c	3.8 ± 0.3i	8.9 ± 0.2b	9.0 ± 0.5a
C18:1	56.8 ± 0.2b	59.5 ± 0.1a	59.7 ± 0.1a	52.2 ± 0.2f	55.5 ± 0.5d	55.8 ± 0.1c	49.9 ± 0.4g	52.7 ± 0.1f	53 ± 0.1e
C18:2	22.2 ± 0.2b	17.5 ± 0.1d	17.8 ± 0.1c	23.9 ± 0.4a	16.2 ± 0.2f	16.4 ± 0.1f	23.9 ± 0.5a	17.0 ± 0.1e	17.4 ± 0.1d
C18:3	4.3 ± 0.3f	2.8 ± 0.1i	3.0 ± 0.1h	8.2 ± 0.2b	4.2 ± 0.1g	4.5 ± 0.2e	11.4 ± 0.2a	6.0 ± 0.1d	6.2 ± 0.1c
ω6:ω3	5.2:1 c	6.2:1a	5.9:1b	2.9:1f	3.8:1d	3.6:1e	2.1:1h	2.8:1fg	2.8:1g

‏*Treatments indicated as olive: sesame: flaxseed, respectively. Different letters represent significant differences (*p*<0.05). All values are the mean of three replicates ± standard deviation of the mean.

Fatty acid composition of oils can change during storage. Results shown in Table 2 confirm that there was a significant change (p< 0.05) during 90 days storage of oil samples. There was a significant decrease (p< 0.05) in 18:2 and 18:3. However, this decrease could not increase the ω_6_:ω_3_ ratio beyond optimal levels. It has been previously reported that changes in fatty acid composition of olive oil occurs during storage.^17^

Vegetable oils generally are kept at two different temperatures; in refrigerator (4°C) or in ambient conditions (24-25°C). Storage of these oil mixtures for up to 90 days at either of these temperatures caused significant changes on fatty acid composition.

Addition of flaxseed oil, which is known as highly-unsaturated and unstable oil but with high nutritional quality, to the mixture of olive and sesame oils, had a positive nutritional effect on the fatty acid profile by increasing of ω_3_ fatty acid ratio. Mixtures of oils with flaxseed oil had excellent oxidation stability during 90-days of storage.

### Total phenolic compound

The amount of phenolic compounds is an important quality factor in oils because of their role in oxidation stability, nutritional and organoleptic qualities.^18^ Olive oil is distinguished from the other vegetable oils by having a high content of phenolic compounds.^19^ Sample 65:30:5 had the highest TPC among the treatments, which is related to the higher amount of olive oil in the blend.

TPC of the samples showed a significant (p< 0.05) slow decrease during the storage until the 60^th^ day (Table 3). This decrease was caused by oxidation and hydrolytic activities which occur because of the effects of temperature, oxygen and enzymes during storage.^20^ On the 90^th^ day TPC increased significantly (p< 0.05) in all samples, which may come from the breakdown of the complex phenolic compounds in the oils to simple phenols. These simple phenolic compounds give a more intense colour with the Folin Ciocalteu reagent.

**Table 3:** Total phenolic compounds (mg caffeic acid/kg of oil) of the three oil blends during 90 days storage at different temperatures.

**Day**	**1**		**30**		**60**		**90**	
Sample*	**4°C**	**25°C**	**4°C**	**25°C**	**4°C**	**25°C**	**4°C**	**25°C**
65:30:5	1652 ± 12.5 e	1652 ± 12.58 e	1611 ± 8.0 f	1448 ± 12.5 h	1568 ± 12.5 g	1423 ± 7.02 hi	1915 ± 10.5 c	2093 ± 60.2 a
65:30:5	1402 ± 7.63 i	1402 ± 7.63 i	1351 ± 9.0 j	1201 ± 9.53 k	1318 ± 7.63 j	1098 ± 12.5 m	1893 ± 11.5 c	2006 ± 10.0 b
65:30:5	1210 ± 10.0 k	1210 ± 10.3 k	1160 ± 8.5 l	1048 ± 7.21 n	1043 ± 15.2 n	983 ± 15.2 o	1798 ± 12.5 d	1983 ± 15.2 b

All values are the mean of three replicates ± standard deviation of the mean. Different letters represent significant differences (*p*<0.05). *For treatments see Table 2.

### Stability parameters

Peroxides generated by the oxidation of fatty acids can affect the quality of oils and also food containing them. Oxidation can be promoted by proxidant metals such as iron and copper, temperature, light, sensitizers such as chlorophyll. Olive oil can have very high PV among oils used in this study. Commercially available virgin olive oils can have PV values of up to 15 meqO_2_/kg oil.^21^ However, flaxseed oil can also oxidize very fast and can have very high PV because they contain high amounts of polyunsaturated fatty acids.

In this study, all samples showed a significant increase (p<0.05) in PV, after 3 months storage (Table 4). As expected, oil samples kept at room temperature had higher PV than the samples stored in a refrigerator. Oxidation is retarded at low temperature.

Also, oil blends with a higher amount of flaxseed oil showed a greater increase in PV (Table 4) which comes from the higher amounts of fatty acids in this oil.

The sample containing 5% flaxseed oil had the highest PV at the early stages of storage but during storage, it was more stable and oxidized less (Table 4). This is related to the high amount of olive oil and low amount of flaxseed oil in the first treatment. Olive oil had high PV but it is stable and does not oxidize as fast as flaxseed oil.

Temperature had a large effect on the PV of the samples. Oxidation at low temperature was almost two times less than that at high temperature (Table 4). Only the 55:30:15 ratio mixture had a higher PV (16 meqO_2_/kg) after storage at room temperature. It was observed that blends containing high amounts of flaxseed oil could be stored at low temperatures for a long period of time.

**Table 4:** Peroxide values (meq O2/kg oil) of the three oil blends during 90 days storage at different temperatures.

**Day**	**1**		**30**		**60**		**90**	
Sample*	**4°C**	**25°C**	**4°C**	**25°C**	**4°C**	**25°C**	**4°C**	**25°C**
65:30:5	5.23 ± 0.25 k	5.23 ± 0.25 k	5.93 ± 0.20 j	6.77 ± 0.25 hi	6.40 ± 0.17 ij	9.47 ± 0.15 d	6.83 ± 0.40 hi	10.97 ± 0.15 c
65:30:5	4.83 ± 0.30 kl	4.83 ± 0.30 kl	6.10 ± 0.26 j	8.90 ± 0.20 e	7.00 ± 0.26 h	9.83 ± 0.15 d	8.10 ± 0.26 f	13.63 ± 0.15 b
65:30:5	4.63 ± 0.30 l	4.63 ± 0.30 l	6.77 ± 0.20 hi	9.40 ± 0.26 d	7.57 ± 0.20 g	11.10 ± 0.36 c	9.57 ± 0.20 d	16.00 ± 0.36 a

All values are the mean of three replicates ± standard deviation of the mean. Different letters represent significant differences (*p*<0.05). *For treatments see Table 2.

Lipid oxidation involves the formation of hydroperoxides as primary oxidation products which are unstable and may break down to a variety of volatile and nonvolatile compounds as secondary products. Secondary products are responsible for off flavors and some of them can be toxic. Secondary products are determined by measuring the p-anisidine content.^22^

Generally, there were no significant increases in AnV until 30 days of storage, but after that it increased in all samples (Table 5). Increase in AnV of oils stored at room temperature was greater than that in the oils kept at refrigerator. Samples containing 15% of flaxseed oil had higher AnV, but it was still lower than for a standard range for common with high stability edible oils.^23^

**Table 5:** Anisidine values of oil blends during 90 days storage at different temperatures.

**Day**	**1**		**30**		**60**		**90**	
Sample*	**4°C**	**25°C**	**4°C**	**25°C**	**4°C**	**25°C**	**4°C**	**25°C**
65:30:5	3.00 ± 0.05 i	3.00 ± 0.05 i	3.00 ± 0.3 i	3.00 ± 0.02 i	3.00 ± 0.02 i	3.50 ± 0.04 g	3.38 ± 0.04 g	3.86 ± 0.05 ef
65:30:5	2.00 ± 0.02 k	2.00 ± 0.02 k	3.00 ± 0.04 i	2.99 ± 0.08 i	3.10 ± 0.01 hi	3.69 ± 0.08 f	4.01 ± 0.06 de	4.25 ± 0.05 c
65:30:5	3.00 ± 0.02 i	3.01 ± 0.02 i	3.00 ± 0.03 i	3.00 ± 0.02 i	3.21 ± 0.03 h	4.03 ± 0.07 d	4.68 ± 0.07 b	6.62 ± 0.04 a

All values are the mean of three replicates ± standard deviation of the mean. Different letters represent significant differences (*p*<0.05). *For treatments see Table 2.

### Schaal test

**Figure 1:**
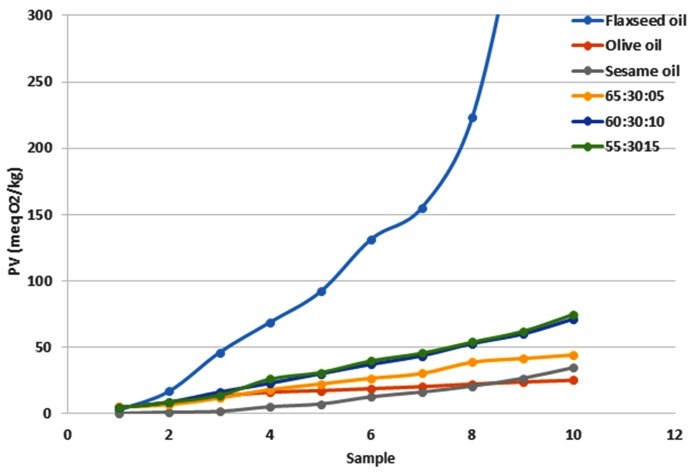
Peroxide value (meq O_2_/kg) of pure olive, sesame and flaxseed oil and their mixtures following an oven test (65°c). For treatments see Table 2.

PV results of the oil blends stored at 65°C for 10 days showed that olive oil is more stable than sesame and flaxseed oils. Flaxseed oil had the lowest stability which has also been previously reported.^24^ Oil blends had a significant differences in PV obtained using the Schaal test (Figure 1). As the amount of flaxseed oil was increased in the oil blends the oxidation stability of the oil blends decreased.

## Conclusion

Incorporating sesame and olive oils with flaxseed oil led to improvement in oxidative stability parameters during storage and also had positive effect on nutritional quality of final product. Oil blends with 10 and 15% of flaxseed oil showed the optimal ratio of essential fatty acids but considering all quality parameters, oil blends with 10% flaxseed oil had the highest oxidation stability. This study illustrates that using flaxseed oil in vegetable oil blends can yield an effective level of bioactive compounds, balanced ω_6_:ω_3_ ratio and suitable stability.

## Acknowledgments

This research was financially supported by Tabriz University of Medical Sciences.

## Ethical Issues

Not applicable.

## Conflict of Interest

The authors have no conflicts of interest to declare.
